# Deciphering the Role of Endolysosomal Ca^2+^ Channels in Immunity

**DOI:** 10.3389/fimmu.2021.656965

**Published:** 2021-04-27

**Authors:** Abeer F. Alharbi, John Parrington

**Affiliations:** ^1^ Department of Pharmacology, University of Oxford, Oxford, United Kingdom; ^2^ Pharmaceutical Sciences Department, College of Pharmacy, King Saud Bin Abdul-Aziz University for Health Sciences, Riyadh, Saudi Arabia

**Keywords:** endolysosomal, calcium, Ca^2+^ signals, immunity, innate response, adaptive response

## Abstract

The role of endolysosomal Ca^2+^ signalling in immunity has been a subject of increasing interest in recent years. Here, we discuss evolving knowledge relating to the contribution of endolysosomal Ca^2+^ channels that include TPCs, TRPMLs, and P2X4R in physiological processes related to innate and adaptive immunity—including phagocytosis, inflammation, cytokine/chemokine release, dendritic, natural killer, and T cell activation and migration—and we underscore the paucity of clinical studies in this field. Emerging biomedical and translational data have led to important new insights into the critical roles of these channels in immune cell function and the regulation of innate and adaptive immune responses. The evolving immunological significance of endolysosomal Ca^2+^ signalling warrants further investigations to better characterize the roles of these channels in immunity in order to expand our knowledge about the pathology of inflammatory and autoimmune diseases and develop endolysosomal Ca^2+^ channels as viable biomarkers and therapeutic and preventive targets for remodelling the immune response.

## Introduction

Innate and adaptive immunity are two fundamental components of the immune system. The cross-talk between innate and adaptive responses is important in maintaining a functional immune system in order to protect the individual against foreign substances such as allergens, toxins, tumour cells, bacteria, and viruses. The innate immune system involves monocytes, macrophages, dendritic cells, mast cells, basophils, neutrophils, eosinophils, and natural killer cells; whereas the adaptive immune system is composed of B cells and T cells. Several studies have indicated that intracellular Ca^2+^ signalling is critical to maintaining various immune cell functions (1–3) and attributed the development of multiple autoimmune and inflammatory diseases to Ca^2+^ dysregulation (4, 5).

**Figure 1:**
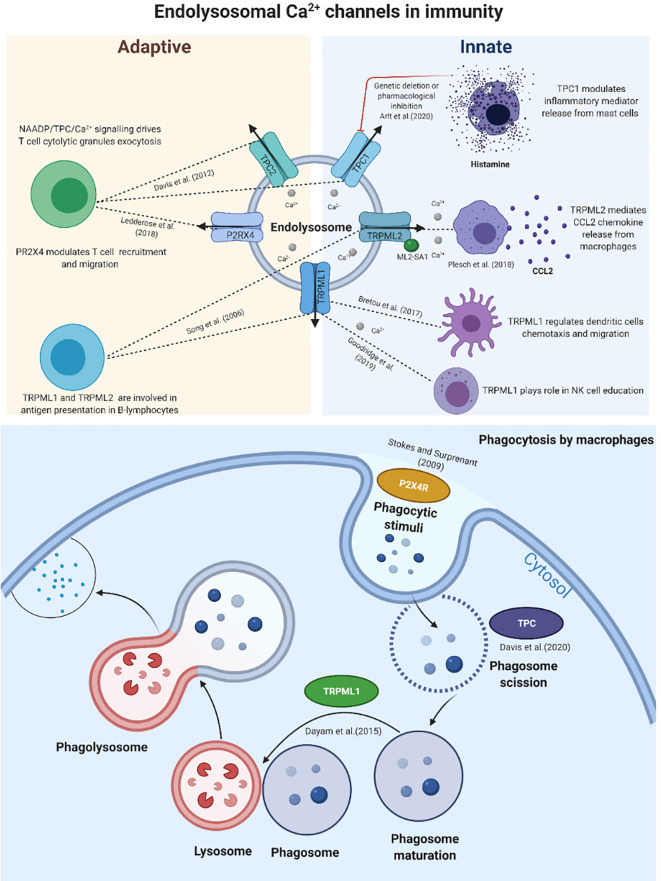
Schematic representation of the main calcium endolysosomal Ca^2+^ channels involved in immunity. The evolving contributions of TPCs, TRPMLs and P2X_4_R in innate and adaptive immune responses and their vital roles in various stages of phagocytosis.

Ca^2+^ signalling mediated by endolysosomal channels is emerging as a player in processes related to immune cell functions such as phagocytosis; the release of inflammatory mediators; antigen presentation; inflammation; cellular trafficking; and T cell migration. Endo-lysosomal Ca^2+^ channels are localized in early, late, and recycling endosomes, lysosomes, and autophagosomes. They are comprised of two-pore channels (TPCs, also known as TPCNs); transient receptor potential cation channels; mucolipins (TRPML); and the P2X4 ATP-activated cation channel. A significant contribution of endolysosomal Ca^2+^ signalling has been demonstrated in phagocytosis, which is a vital physiological process in cellular immunity mediated by TPCs, TRPML1, and P2X4R (6, 7). TRPML2 is an endolysosomal Ca^2+^ channel that has been shown to have direct roles in the release of chemokine/cytokine (8). Additionally, TPC1 is an endolysosomal Ca^2+^ channel that has been reported to be involved in the development of the immune response and the release of inflammatory mediators (9). Although it has become clear that endolysosomal Ca^2+^ signals play pivotal roles in health and disease, the complex dynamics underlying the regulation of Ca^2+^ signalling via endolysosomal channels and the involvement of these in physiological processes related to immunity have remained elusive. Here, we highlight the emerging roles of endolysosomal Ca^2+^ channels in various physiological processes related to immunity (as shown in **Figure 1** and **Table 1**). Our intent is to reveal their potential as key pieces in a puzzle that will help increase understanding of the pathophysiology of autoimmune and inflammatory disorders and develop endolysosomal Ca^2+^ channels as targets for future immunotherapy.

**Table 1:** Some experimental evidence supporting endolysosomal Ca^2+^ signalling involvement in physiological processes attributed to immunity.

Immune system	Endolysosomal Ca^2+^ channel	Process related to immunity	*In vitro*/*in vivo*	Ref.
**Innate immunity**				
Macrophages	TPCs	Phagocytosis	RAW 264.7,BMDM of WT, TPC1KO, TPC2KO, TPDKO and TRPML-1KO mice	(6)
	TRPML1			
	P2X_4_R		Human alveolar, THP-1, NR8383, J774, mouse peritoneal, and RAW264	(7)
	TRPML2	CCL2 chemokine release	BMDM of WT and TRPML-2KO mice	(8)
Dendritic cells	TRPML1	DC chemotaxis, and migration	BMDCs of WT and TRPML1 KO mice	(10)
	P2X_4_R	Priming dendritic cells for Th2 inducing IL-1ß secretion	BMDCs of WT and P2RX_4_ KO mice	(11)
Mast cells	TPC1	Inflammatory mediator release (histamine)	WT and TPC1KO mice	(9)
NK cells	TRPML1	NK cell education	NK cells sorted from PBMC	(12)
**Adaptive immunity**				
B-cells	TRPMLs (TRPML1 and TRPML2)	B-cell antigen presentation	DT40 B-lymphocytes	(13)
T-cells	P2X_4_R	T-cell recruitment and migration	Primary T cells and Jurkat T cells	(14)
	TPCs (TPC1 and TPC2)	T cell cytolytic granule exocytosis	Jurkat 1G4 cells	(15)

## TPCs in Phagocytosis, Inflammatory Response, and Virus Trafficking

The two-pore channels (TPCs) are present as two isoforms in mammals—TPC1 and TPC2. Debate continues as to whether TPCs are primarily Ca^2+^ or Na^+^ channels (16). Data from several studies suggest that TPCs behave differently in different biological contexts. They can trigger Ca^2+^ or Na^+^ release upon binding to second messengers: nicotinic acid adenine dinucleotide phosphate (NAADP), acts directly or indirectly to release Ca^2+,^ and phosphatidylinositol 3,5-bisphosphate [PI (3,5)P_2_] to release Na^+^ (17–20). Phagocytosis by macrophages is a physiological process initiated by our innate immune system as the first line of defines against both pathogens (bacteria, toxins, viruses) and tumour cells. Lysosomes are multifunctional organelles and play vital roles in phagocytosis, particularly in the late stages of phagosome maturation (21). A recent study by Suresh et al. (22) has uncovered the role of the tubular state of lysosomes in phagocytosis, which is known to modulate processes related to immunity, such as antigen presentation. The study found that lysosome tubular states mediate phagocytosis and enhanced phagosome-lysosome fusion in RAW 264.7 cells (an *in vitro* model of murine macrophages) (22). Another recent study by Freeman et al. suggested the possibility that TPC2 acts as a regulator of the lysosome tubulation process (23). The study showed that TPC2 overexpression drives lysosome tubulation in a mechanism involving phosphatidylinositol 3,5-bisphosphate activation (23). Additionally, TPC1 and TPC2 expression at the mRNA level was found to be significantly upregulated in bone marrow-derived macrophages compared to mouse embryonic fibroblasts (23). The participation of endolysosomal Ca^2+^-mediated phagosome-lysosome fusion was implicated in the maturation of the phagosome phase, where the phagosome fused with the lysosome, ultimately becoming a phagolysosome, which is a fundamental step of phagocytosis. Recently, Davis et al. (6) identified a role for NAADP evoked TPC-endolysosomal Ca^2+^ signalling from the nanodomains involving calcineurin activity and dynamine 2 activation in macrophages at the scission of phagosomes from the plasma membrane stage of phagocytosis for small and large particles (6), which suggests that TPC1 or TPC2 may act as downstream regulators of phagocytosis in macrophages.

Previously, Davis et al. deciphered the biological significance of NAADP/TPC/Ca^2+^ signalling in T cell biology. They found that NAADP-mediated Ca^2+^ release is a significant pathway that drives T cell cytolytic granule exocytosis (15). Recently, Elisabeth et al. (2020) reported for the first time that endolysosomal Ca^2+^ signals via TPC1 mediate the development of the immune response by triggering the release of inflammatory mediators in a mechanism involving Ca^2+^ cross talk between TPC1-mediated and endoplasmic reticulum (ER) Ca^2+^ stores (9). In the *in vivo* TPC1KO murine model, systemic anaphylaxis was exaggerated, manifested by a profound drop in body temperature compared to WT mice (9). The study also found that TPC1 modulation either by genetic deletion or by pharmacological inhibition by *trans*-Ned-19 augmented mastocyte degranulation and evoked the release of inflammatory mediator (histamine) from mast cells (9), which are tissue-resident cells of the immune system that play a role in inflammatory and allergic reactions. The number and the size of the mastocytes were significantly attenuated in the TPC1-deficient murine model compared to WT controls (9). The cellular mechanisms underlying the regulation of TPC1-mediated endolysosomal Ca^2+^ signals in the development of inflammatory and allergic reactions warrants further investigation to aid the development of new drugs for the treatment of anaphylaxis and allergic hypersensitivity.

The NAADP/TPC/Ca^2+^ signalling pathway has been shown to play an important role in virus trafficking. Gunaratne et al. showed that TPC (TPC1 and TPC2) knockdown hampered Middle East Respiratory Syndrome coronavirus (MERS-CoV) infection in human embryonic kidney 293 (HEK293) cells (24). Ca^2+^ signalling via TPCs (involving TPC1 and TPC2) regulates Ebola virus entry and plays a significant biological role in virus trafficking and preventing the infection (25). Pharmacological inhibition or genetic knockout of TPCs diminished the capacity of the Ebola virus to infect cells in *in vitro* or *in vivo* models (25). The candidacy of these channels as druggable targets for future antiviral therapy is supported by the availability of FDA-approved drugs, such as dopamine antagonists (e.g. fluphenazine and pimozide) and selective oestrogen receptor modulators (including raloxifene, clomiphene, and tamoxifen) that inhibit TPC function and hinder Ebola virus-like particle entry into HeLa Kyoto cells *in vitro* (26). Functional characterisation of fluphenazine and raloxifene revealed that they block TPC2 activity by decreasing the channel opening time (26). The SARS-CoV2 outbreak led to a revisiting of the role of endolysosomal Ca^2+^ signalling, particularly via TPCs, in virus trafficking and infectivity. Recent evidence has shown that TPC2, phosphatidylinositol 3-phosphate 5-kinase (PIKfyve), and cathepsin L regulate SARS-CoV-2 entry in an *in vitro* model (HEK 293/hACE2 cells) (27). Similarly, Clementi et al. found that inhibition of TPC2 function via pharmacological means by naringenin or knockdown by siRNA attenuated SARS-CoV2 infection *in vitro* (28).

Despite the growing evidence linking TPC/Ca^2+^ signalling to physiological processes attributed to immunity, research in this area is still in its infancy. Further investigations utilizing biomedical (*in vitro* and *in vivo*) and clinical models will decode the role of TPC/Ca^2+^ signalling in immunity and will contribute to advancing our knowledge regarding the roles of this signalling pathway in the pathogenesis of immune system diseases and might lead to the development of therapeutic agents to treat or prevent diseases related to the immune response.

## TRPMLs in Phagocytosis, Antigen Presentation and Chemokine/Cytokine Release

Transient receptor potential cation channels (TRPMLs; mucolipins) are a subfamily of the TRP channel family, and composed of TRPML1, TRPML2 and TRPML3 in mammals; they are localized in the endolysosomal compartments (29). TRPMLs play a significant role in endolysosomal biology, specifically, endolysosomal trafficking that leads to autophagy (30). Notably, the roles of TRPMLs (particularly TRPML1 and TRPML2) in physiological processes related to immune cell functions are evolving (31, 32), underscoring the importance of fully characterizing the biological and clinical functions of these channels in the immune system. Song et al. (33) reported the first evidence of the interconnection between TRPMLs (TRPML1 and TRPML2) and B-cell antigen presentation in vertebrates (33). The TRPML1-mediated Ca^2+^ signalling pathway has been implicated in phagocytosis (6, 13, 34). TRPML1 acts as a regulator of phagosome maturation, FYVE finger-containing phosphoinositide kinase (PIKfyve), and (PI(3,5)P_2_)-mediated Ca^2+^ signals via TRPML1-triggered phagosome-lysosome fusion (13). A previous study implicated (PI(3,5)P2)/TRPML1/Ca^2+^ signalling as a modulator of phagocytosis by regulating focal exocytosis, which is significant for phagosome biogenesis (34). Recently, knockout of TRPML1 was shown to attenuate the phagocytosis of large particles in murine bone marrow derived macrophages (BMDMs), indicating that lysosomal Ca^2+^ release via TRPML1 is necessary for large target phagocytosis (6). TRPML1/Ca^2+^ signalling is involved in lysosome tubulation (35); this process plays roles in phagocytosis and antigen presentation. Recently, Goodridge et al. (36) identified a pivotal role for lysosomal Ca^2+^ release via TRPML1 as a meditator of the natural killer (NK) cell function (36). Previous studies demonstrated that TRPML1 lysosomal Ca^2+^ signals were involved in dendritic RNA transportation through the modulation of Toll-like receptor 7 (TLR7) signalling (12). Additionally, the Ca^2+^ signalling mediated by TRPML1 regulates two important dendritic cell functions involving migration and chemotaxis (37). These findings highlight the evolution of TRPML1 as a modulator of innate and adaptive immune cell functions; thus, it warrants further investigation to reveal the molecular mechanisms of TRPML1 in immunity via *in vitro* and *in vivo* models. TRPML2/Ca^2+^ signalling modulates chemokine (C-C motif) ligand 2 (CCL2; also known as monocyte chemoattractant protein 1 (MCP1)) release from macrophages (8) and acts as a key regulator of monocyte and macrophage infiltrations and migration (10). Consequently, Ca^2+^ signalling via TRPML2 modulates the inflammation by regulating the release of CCL2; this may serve as a viable therapeutic target for patients with inflammatory diseases. In addition to the involvement of TRPML2 in chemokine release, it was shown that TRPML2-evoked endolysosomal Ca^2+^ signalling plays a role in viral trafficking (38). To our knowledge, there is a lack of molecular evidence characterising the role of TRPML3 in immunity. Although the evolving body of evidence highlights that endolysosomal Ca^2+^ signals mediated by TRPMLs play important roles in innate and adaptive immunity, further studies are required to decipher the precise mechanisms underlying the physiological processes of these channels in immunity, from chemokine/cytokine release to antigen presentation.

## P2X_4_ R in Phagocytosis, Inflammation, Cytokine Release and Involvement in T Cell Migration

The P2X_4_ receptor belongs to the purinergic receptor family, is involved in ATP-evoked Ca^2+^ release, and is localized to the endolysosomal system (39). P2X_4_R-mediated Ca^2+^ signalling is recognized as a key mediator in inflammation and neuropathic pain (40–42). Recent studies have continued to shed light on the roles of P2X_4_R in physiological processes related to immunity. Upregulation of P2X_4_R protein expression was observed in the early stages of phagocytosis (with initial phagocytic stimuli) in alveolar macrophages (7), which suggested the involvement of P2X_4_R in phagocytosis. As discussed earlier concerning the roles of TPCs and TRPMLs in phagocytosis, we speculate that endolysosomal ion channels dynamically communicate at a molecular level to ultimately mediate phagocytosis; additionally, these channels have distinctive roles in different phases of this process. P2X_4_R mediates allergen-induced airway inflammation through the regulation of priming dendritic cells for T helper 2 (Th2), inducing IL-1ß (Interleukin 1 beta) secretion (43). Similarly, P2X_4_R was found to modulate the P2X7 receptor-mediated release of two pro-inflammatory cytokines, IL-1ß and IL18 (Interleukin-18), which mediate inflammation in murine bone marrow-derived dendritic cells (BMDCs) (11). P2X_4_R-deficient mice exhibited protective effects against ischemic acute kidney injury compared to WT mice; this effect was studied at the molecular level and linked to P2X_4_R-augmented ischemic acute kidney injury via a mechanism involving the activation of NLRP3 (NLR family pyrin domain containing 3) in inflammasome signaling (44). Inflammasome is a multiprotein complex that plays a fundamental role in inflammation of innate immune cells through the activation of caspase 1, which is responsible for cleavage of the precursor forms of two important inflammatory mediators, IL-1ß and IL18, into biologically active cytokines (44). Further research is required to determine the complex interplay between P2RX_4_, inflammasome, and the release of IL-1ß and IL18. Pharmacological inhibition of P2RX_4_ via 5-BDBD (5-(3-bromophenyl)-1,3-dihydro-2H-benzofuro[3,2-e]-1,4-diazepin-2-1) hampered T-cell migration (45). T-cell migration is a critical step in T-cell function. Additionally, Ledderose et al. (44) have provided substantial further evidence to confirm this finding. With an *in vivo* mouse model, they found that pharmacological modulation of P2X_4_R by 5-BDBD resulted in the rejection of lung transplants by impairing T-cell recruitment in allograft tissue (45). Overexpression of P2X_4_R was detected in CD4^+^ T cells from peripheral blood and adipose tissue in obese, healthy subjects, indicating a possible role for P2X_4_R in chronic inflammation associated with obesity (46).

Overall, these findings underline the immunological significance of P2X_4_R in innate and adaptive immunity and warrant further investigations to biologically and clinically characterize the multi-functional role of P2X_4_R in immunity. This may further the development of immunomodulators to treat inflammatory diseases and prevent graft rejection and transplantation complications.

## Clinical Investigations of Endolysosomal Ca^2+^ Channels in Disorders Related to the Immune System

Although there is a growing scientific interest in the role of endolysosomal Ca^2+^ channels in immunity, there is a paucity of studies that categorize these channels clinically. Recently, a genome-wide association study in the Han Chinese population identified TPCN2 as one of four gene signatures attributed to systemic lupus erythematosus (SLE) susceptibility, which is characterized as a chronic autoimmune disease (14). Significantly overexpressed P2X_4_ at the protein level was discovered in tissues of patients with hepatitis C virus-induced hepatocellular carcinoma compared to non-hepatitis C virus-induced hepatocellular carcinoma (47). This finding raises a clinical question regarding the possibility of targeting P2X_4_ to modulate the immune response that contributes to hepatitis C virus-induced hepatocellular carcinoma, which warrants further investigation to understand the role of P2X4 in hepatitis C virus-induced hepatocellular carcinoma pathology.

## Closing Remarks and Future Perspectives

An evolving body of evidence continues to uncover the function of endolysosomal Ca^2+^ signalling in innate and adaptive immune cell responses. It has become clear that endolysosomal Ca^2+^ channels, mainly TPC2 and TRPML-1, serve a critical role in phagocytosis at a global level, with distinctive roles at different stages of the phagocytic process. Importantly, TRPML-2 and P2X_4_R are implicated in modulating chemokine and cytokine release and consequently their effect on inflammation; however, the precise mechanisms underlying the action of these channels remain elusive and require further investigation to define a specific upstream or downstream target to overcome problems posed by the ubiquity of Ca^2+^ signals in our cells and to modulate the innate immune response. Recent studies shed light on the roles of TRPML-1 and P2X_4_ in adaptive immune cell function and raise questions regarding their candidacy as valuable targets for modulation of adaptive immune responses. Regardless of the paucity of clinical evidence, GWAS revealed the potential applications of TPC2 as a biomarker in the definition of SLE susceptibility in the Chinese population and warrants validation in prospective cohorts of a diverse population. Despite the exploratory nature of the evidence highlighting the role of endolysosomal Ca^2+^ signalling in various processes related to immunity, this mini-review offers some insights into the pivotal roles of these channels in the specific mechanisms of innate and adaptive immunity that lead to inflammation and disorders related to the immune system. Additionally, it raises questions regarding the clinical utility of these channels as biomarkers or immunotherapy targets to modulate innate and adaptive immune responses.

## Author Contributions

AA has contributed to the mini-review-research question, data collection, and interpretation, and written the manuscript. JP has revised the manuscript. All authors contributed to the article and approved the submitted version.

## Conflict of Interest

The authors declare that the research was conducted in the absence of any commercial or financial relationships that could be construed as a potential conflict of interest.
